# Stability of Kinect for range of motion analysis in static stretching exercises

**DOI:** 10.1371/journal.pone.0200992

**Published:** 2018-07-24

**Authors:** Fatemeh Mortazavi, Ali Nadian-Ghomsheh

**Affiliations:** Cyber Space Research Institute, Shahid Beheshti University, GC, Tehran, Iran; Berner Fachhochschule, SWITZERLAND

## Abstract

Physical rehabilitation aims people with physical impairments to enhance and restore their functional ability. The Microsoft Kinect v1 and v2 technologies apply depth information and machine vision techniques to generate 3D coordinates of a set of anatomical landmarks on the human body regarded as Kinect joints. Trigonometry relationship between Kinect joints can be used to extract body Range of Motion (ROM). The purpose of this study was to evaluate stability of Kinect for ROM measurement during static stretching exercises. According to the literature, the stability of Kinect in static exercises has been reported to a limited extent. 13 healthy men participated in this study and performed 5 exercises in 2 different distances from the cameras. Exercises were recorded by Kinect v1 and Kinect v2, concurrently. The stability of Kinect was also evaluated for 5 ROMs including: elbow flexion, shoulder abduction, wrist pronation, wrist flexion, and wrist ulnar deviation. Maximum and average joint displacement errors were used for stability analysis. Results showed that Kinect v2 is more stable compared to Kinect v1. Kinect v2 joints showed displacement error of more than 15 mm for wrist. For the other joints, Kinect showed an average displacement error of less than 10 mm.

## Introduction

One of the important objectives of physical rehabilitation is to optimally restore the lost motor functionality resulted from disabling impairment or disease. The goal for the patients is to regain the ability needed for daily activities. Repetitive stretching exercises aim to improve the functionality of joints and thus keep joints flexible. ROM is the measurement of movement around a specific joint in a certain direction and is used to evaluate the flexibility of joints. Traditional physical therapy programs evaluate ROM with a goniometer [1]. In recent years, various technologies such as robotics and virtual reality have been used in a wide range of rehabilitation applications, including ROM measurement [2].

Introduction of low-cost marker-less motion capture systems have attracted many researches to explore and evaluate this technology for physical rehabilitation. Among commercial depth cameras, Kinect, due to its low-cost and the accompanying Software Development Kit (SDK) has gained more attention [3]. The SDK is a collection of programming libraries that allows for rapid application development. Kinect was released by Microsoft cooperation in 2011. The second generation of Kinect, i.e. Kinect v2 was later released in 2013 with enhanced camera and tracking features. Both cameras apply an infrared imaging system to capture the scene depth. A pattern recognition algorithm is also implemented to extract a set of anatomical landmarks on the human body regarded as Kinect joints. The number of anatomical landmarks provided by Kinect v1 is 20 while for Kinect v2 this number is increased to 25. Regarding the imaging technology, Kinect v1 uses structured light sensing and Kinect v2 implements the time of flight method for capturing the scene depth. Furthermore, Kinect v2 provides a depth image with higher resolution and lenses with wider field of view. The pattern recognition algorithm that is used for obtaining Kinect joints is reported in Shotton et al [4]. This algorithm was trained on a large dataset for recognizing in-game human interactions. However, low-cost and easy software development has attracted researchers to evaluate Kinects applications in other human-centered applications. When Kinect is applied in a context other than gamming, it is important to examine the accuracy and stability of this technology for the specific application. Kinect is mostly evaluated for applications such as ROM assessment, exercise monitoring, and motivation promotion.

Motivation promotion using Kinect is mostly achieved by developing computer games. In game based interventions, exercising with a computer game can increase the patient’s motivation to complete the assigned tasks. In this methodology Kinect is mostly used to capture the patient’s motion data which is used for controlling game parameters [5,6].

Monitoring and ROM assessment are other applications of the Kinect technology that have been widely studied. These methods are designed to measure the ROM or inspect the overall performance of the patient. The goal of these systems is to quantify the motor function improvement level of patients [7]. Chang et al. [8], described a study that assesses the possibility of rehabilitating 2 young adults with motor impairments using a Kinect. The proposed system measures the upper-limb range of motion. A therapist can use these measurements to specify angles of shoulder flexion, shoulder extension, shoulder external abduction, shoulder external rotation, shoulder internal rotation, and elbow flexion for upper limb rehabilitation. Kitsunezaki et al. [9], designed a real-time ROM measurement system to evaluate walking time and joint angle ranges using Kinect. Results showed that measurable space is limited, 3-dimensional X, Y, Z coordinate data have relatively large differences of 1 cm, and the time difference between Kinect measurements and Vicon system was 0.33 seconds in average.

Although Kinect has shown to be a useful motion capture system for rehabilitation applications, the spatial accuracy of this system should be examined. According to the literature, several studies have been conducted in this context [10,11]. Wang et al. [12], focused on accuracy and performance of Kinect v1 and v2 skeleton tracking system in comparison with a marker-based motion capture system. The motion data of 10 participants performing 12 exercises captured from 3 different viewpoints was used in this study. They reported that joint’s position accuracy in Kinect v2 is superior to Kinect v1 and spatial distribution of Kinect v2 results in smaller number of outliers. Yang et al. [13], measured the depth accuracy of the Kinect v2 depth sensor and reported that the position of the target object observed by three Kinect sensors has 51.22 mm displacement in average. Flexible Action and Articulated Skeleton Toolkit (FAAST), was presented by Suma et al. [14]. They used Kinect for real time feedback in treadmill based training. They compared the position of hands and feet obtained from motion capture and Kinect, and found errors in the range of 5–7 cm. Reither et al. [15], compared the reliability and validity of Kinect v1 and Kinect v2 based on a Video Motion Capture (VMC) system for upper extremity movements. They showed that ROM magnitudes for both Kinects were different from the VMC, but, the patterns of motion were correlated for both devices. Fernandez et al. [11], investigated the precision in the computation of joint angles for Kinect and compared it with the results obtained from an optical motion capture system. Comparisons were made for knee, hip and shoulder joints. Results show that all errors are less than 10°. Lee et al. [16], applied Kinect to measure shoulder ROM in Adhesive Capsulitis (AC). They compared their results with goniometry readings and reported the results based on intra-class correlation coefficient (ICC). The ICC for flexion/abduction/external rotation between goniometric passive ROMs and the Kinect ROMs were 0.906/0.942/0.911. ICC between active ROMs and the Kinect ROMs were 0.864/0.932/0.925. Otte et al. [17], evaluated the accuracy of Kinect v2 sensor for clinical motion analysis against a gold standard motion capture system. They explored the spatiotemporal accuracy of 21 anatomical landmarks captured in a set of six motor tasks and studied the accuracy and repeatability of derived clinical parameters. Signal to noise ratio indicated a large noise behavior in feet and ankles. Most of the derived clinical parameters showed good to excellent absolute agreement and consistency.

In physical rehabilitation, static stretching is an effective method for increasing ROM. It has been suggested that 10 to 30 seconds of stretching is sufficient for increasing flexibility [18, 19]. Reliable ROM measurement using a Kinect device requires stable reading of the joint position in a still pose.

Based on the studies on validity of Kinect in rehabilitation applications, some shortcomings are worth mentioning:

1. Previous studies show correlation between the results of Kinect and those obtained from goniometry or standard motion capture systems. However, the reported accuracies vary significantly among previous studies.
2. Static exercises are widely practiced, however, most of the previous work is concerned with evaluating Kinect during dynamic exercises.
3. Hand tips and thumbs are recognized in Kinect v2. These joints can be used to calculate the range of motion for wrist. Less studies have considered these joints for evaluation.

This study is focused on assessment of stability of Kinect during static exercises. Several stretching exercises were performed by healthy subjects for a fix period of time. The position of joints were recorded with Kinect v1 and Kinect v2. The space distance of a joint from the reference point was considered as joint displacement error. The readings were analyzed using maximum and average displacement errors. In summary this study makes the following contributions:

1. Assessment of ROM during static stretching exercises.
2. The Stability of joints introduced in Kinect v2.
3. The effect of joints displacement error on ROM measurement was investigated.

The dataset used in this study can be downloaded with the following DOI: 10.6084/m9.figshare.5027741.v1. The paper is structured as follows: Section 2 briefly reviews the application of Kinect technology in physical rehabilitation. Section 2 details the methodology of the research. Results are presented in Section 3. Section 4 is dedicated to the descussions and the paper is concluded in section 5.

## Method

### Experimental setup and procedures

13 men (height: 182 ± 8 cm, weight 76.5 ± 10 kg, average age: 25 ranging:24–27) volunteered to participate in this study. All participants were students at Shahid Beheshti University who have signed a written consent form. The recording session was explained for each participant individually and a pictorial tutorial guided the participants through the recording session. The study was approved by the ethics committee at Shahid Beheshti University and was granted the code SBU.ICBS.96/1022 for conduct.

Each participant stood directly facing the Kinect sensors and their anatomic position was the stand position with toes pointed to front. The sensors were placed next to each other and positioned 1m from the ground with the lens perpendicular to the floor. First, each participant performed the specified exercise at the distance of 2m from the camera while holding the required pose for at least 10 seconds. Afterward, they repeated the procedure at the distance of 3m form the camera. These distances were chosen based on previous studies [11]. All the sessions were recorded on November, 2016.

**Fig 1:**
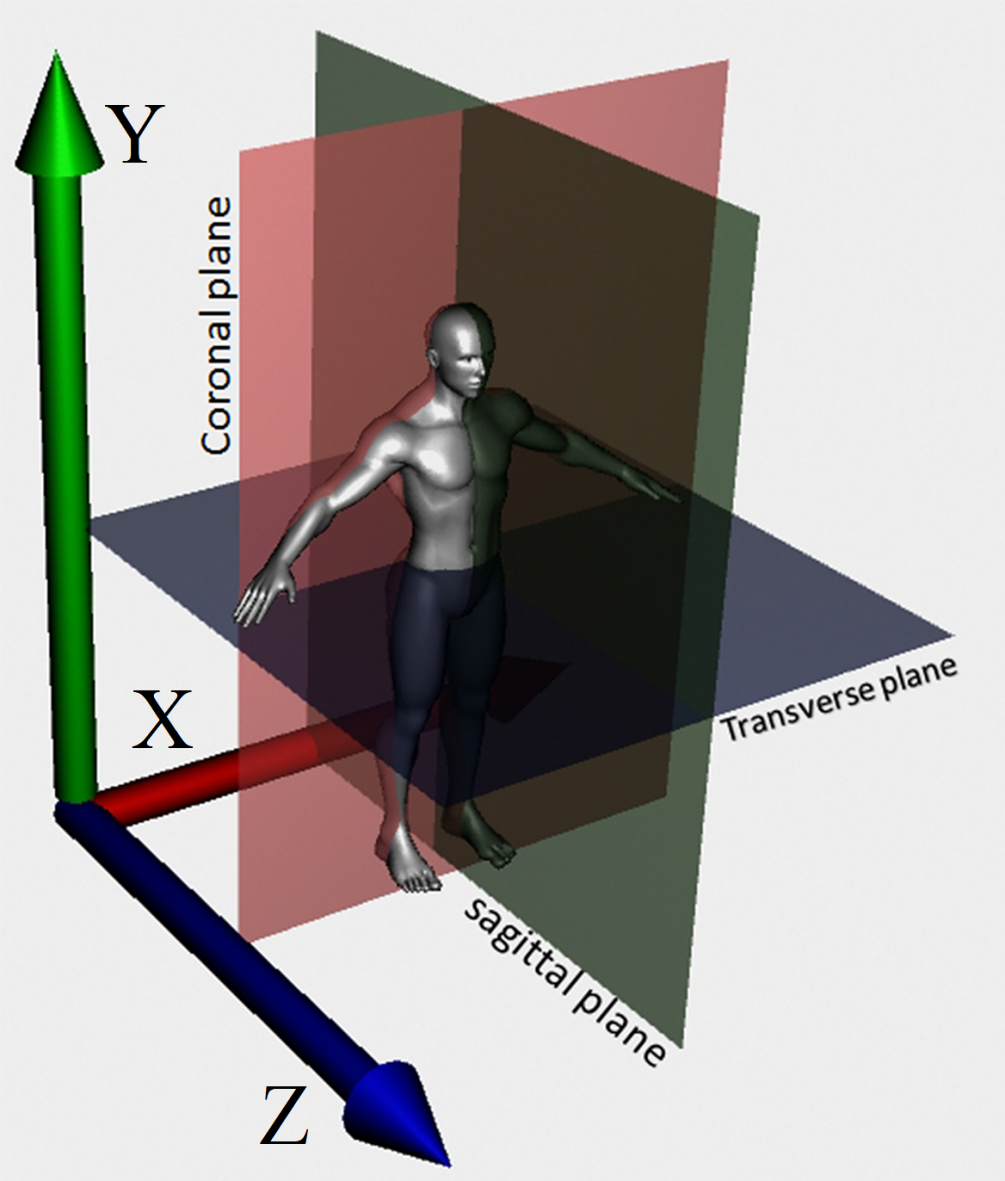
Body tridimensional planes.

Human body movements are tridimensional, allowing the body to move through XY, XZ and YZ space planes [20]. When the body and the planes are projected into the same space, joint movements can be related to these planes. The XY plane, regarded as frontal or coronal plane divides the body into front and back. The YZ plane, called sagittal or vertical plane divides the body into right and left side. XZ is the horizontal or transversal plane which divides the body into up and down portions (Fig 1).

**Fig 2:**
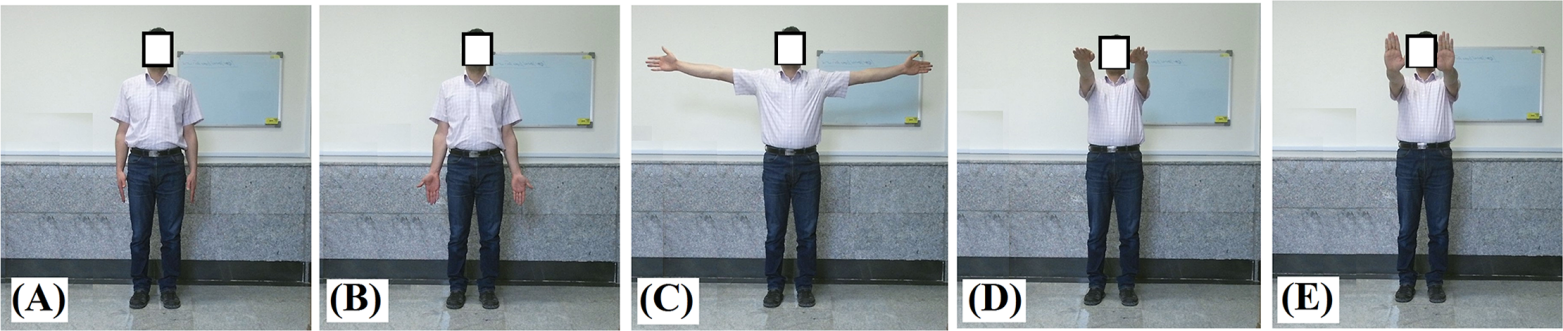
Illustration of exercises (A-E) represent the exercises E1: E5.

According to above explanation, the definition of selected exercises is as follow:

Exercise 1 (E1): subject is in stand position, hands are in coronal plane, elbow is extended, and shoulder is adducted with hand palm facing the body (Fig 2A).
Exercise 2 (E2): subject is in stand position, hands are in coronal plane, elbow is extended, and shoulder is adducted with hand palm facing forward (Fig 2B).
Exercise 3 (E3): subject is in stand position, hands are in coronal plane, shoulder is abducted to 90°, and elbow is extended with hand palm facing forward (Fig 2C).
Exercise 4 (E4): subject is in stand position, arms are in sagittal plane, shoulder flexion is 90°, and elbow is extended with hand palm facing the ground (Fig 2D).
Exercise 5 (E5): subject is in stand position, hands are in sagittal plane, shoulder flexion is 90°, and elbow is extended with hand palm facing forward (Fig 2E).

### Acquisition systems

#### Kinect v1

The Microsoft Kinect is a set of sensors developed as a peripheral device for the Xbox 360 gaming console. It contains a normal RGB camera, an infrared transceiver system, and a multi array microphone. RGB camera delivers the 3 basic color components of the video. The camera operates at 30 frames per second and captures images with 640×480 resolution. Depth sensor consists of an infrared laser projector and an infrared camera. Together, the projector and the camera create a depth map which provides the distance between an object and the camera. The Kinect for windows SDK is a set of libraries that can be used to develop applications on a variety of Microsoft platforms. The Kinect v1 SDK can track 2 users and provide 3D position of 20 joints for each tracked skeleton.

#### Kinect v2

In 2013, Microsoft released the second generation of the Kinect sensor with improved skeleton tracking, facial expression detection, and a higher resolution depth and color images compared to Kinect v1. Kinect v2 is based on the time-of-flight principle. The RGB camera captures color information with resolution of 1920×1080 pixels. The IR camera is used for real-time acquisition of scene depth and also IR data with a 512×424 resolution. The outputs are generated with a frame rate up to 30 Hz. Microsoft Kinect SDK v2.0 provides the skeleton data and enables researchers to develop sophisticated computer-based human motion tracking applications. The SDK for Kinect v2 generates a skeleton frame for up to 6 users, where each skeleton contains 25 joints.

#### Data acquisition

**Fig 3:**
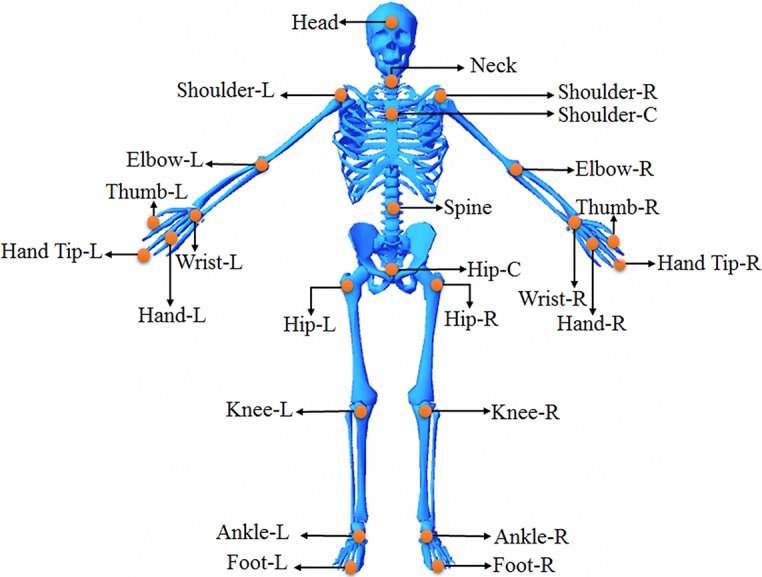
Position and name of Kinect joints. L, R and C represent Right, Left and Central joints, respectively.

For stability analysis, it is important to determine Kinect data streams. When Kinect is in the recording mode, the color and depth image are captured every 32 milliseconds. The Kinect software, tries to detect the human body in the captured image, and if a body was found, 3D position of 20 joints (25 for Kinect v2) are estimated. Each joint is represented by a three dimensional vector (X, Y, and Z) in the coordinate space of Kinect. X, Y, and Z represent the horizontal distance, vertical distance, and depth of the estimated joint with respect to the camera. Each joint is named with respect to its position on the body as shown in Fig 3.

For data acquisition, two Kinect cameras were connected to a single PC running on Windows 10. For this purpose, Kinect v1 connects via USB 2.0 and Kinect v2 connects via USB 3.0 on a separate PCI bus. Skeleton data were recorded using SDK v1.8 for Kinect v1 and SDK v2.0 for Kinect v2. The position of the joints were written into a text file. The timestamp for each frame was also recorded. Each pose was recorded for a period of 10 seconds at 30 frames per second resulting in a minimum of 300 sets of joints positions per record.

#### Data synchronization and pre-processing

It is possible that one of the Kinects drops a frame and the joint sequence become unaligned with respect to time. In order to compare the stability of measured position of joints between two cameras at the same time, data sequences were synchronized using frame timestamps. The skeleton frame is expected every 32miliseconds. If this time exceeds 50 milliseconds, the synchronization process assumes a missing frame. In this case, the equivalent frame captured by the other Kinect was deleted. This procedure was repeated for both Kinect cameras until the timestamp difference between two frames did not exceed the 50 milliseconds threshold. Synchronized data were used for stability analysis. Also, it is possible that Kinect could lose track of some body parts. Thus, invalid data is produced by Kinect which can be regarded as outliers. To resolve this issue, the histogram of joints position in each direction was obtained and values with occurrence probability less than 10% were discarded as outliers.

### Stability analysis

Previous studies have evaluated Kinect for its accuracy. Accuracy is defined as the similarity between ground truth position of joints measured by some standard method and those measured by Kinect [12]. The present study intends to evaluate the stability of Kinect defined as variations of Kinect readings with respect to a reference point in a quasi-still position. Since the variation of measurements was of interest, the average value of the samples per record was used as the reference point:

$${ref}_{testcase}(X_{j})=\frac{1}{F}\sum_{f=1}^{F}X_{j,f}$$ (1)

$${ref}_{testcase}(Y_{j})=\frac{1}{F}\sum_{f=1}^{F}Y_{j,f}$$ (2)

$${ref}_{testcase}(Z_{j})=\frac{1}{F}\sum_{f=1}^{F}Z_{j,f}$$ (3)

A test case specifies an exercise performed at a specific distance by an individual participant captured by one of the Kinects. X, Y, and Z represent horizontal distance, vertical distance and depth of joint *j*, respectively. *F* is the total frames in each test case.

### Measure for stability

Stability of Kinect for ROM measurement was evaluated by inspecting the displacement error of joints with respect to a reference point. Two measures were used in this regard, Sum of Square Error (SSE) and Maximum Displacement Error (MDE). SSE denotes the distance between the position of a joint measured by Kinect and the reference value. SSE is obtained by:

SSEj=1F∑f=1F(Xf,j−Xref,j)2+(Yf,j−Yref,j)2+(Zf,j−Zf,j)2 (4)

Where *f* denotes the index of frame, *j* = 1: 20 (25 for Kinect v2) represents the joints. *X*, *Y*, and *Z* represent the space coordinate of *j*^*th*^ joint in frame *f*.

**Fig 4:**
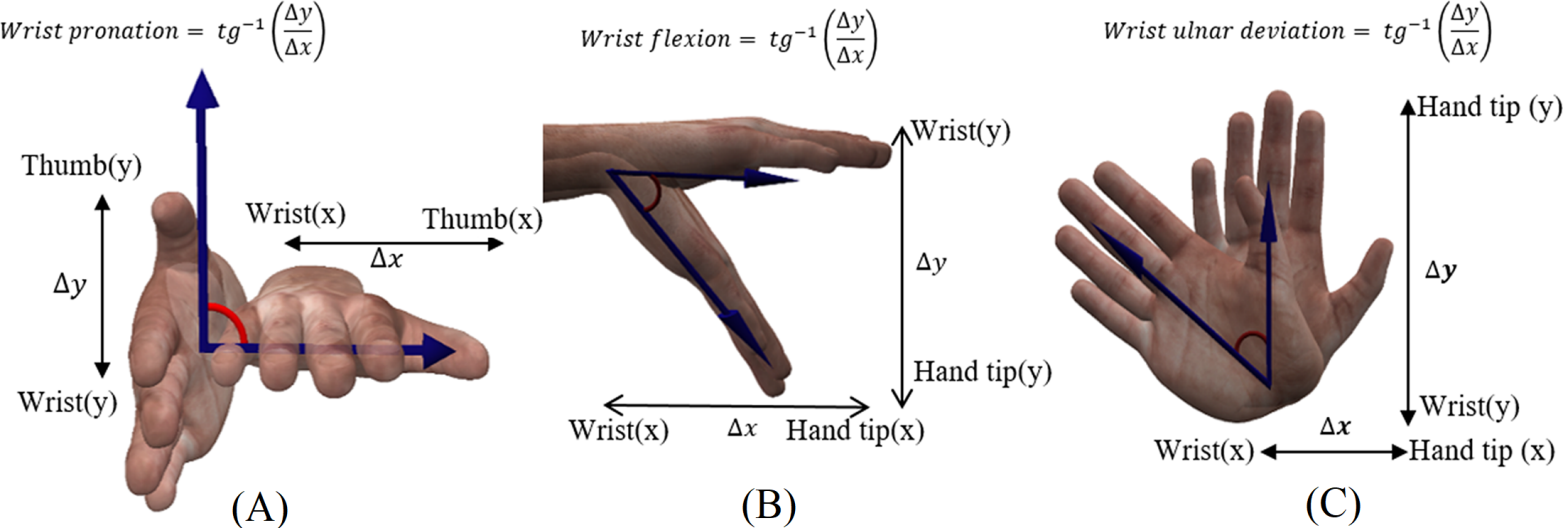
Illustration of (A) wrist pronation, (B) flexion, and (C) ulnar deviations. Formulation for extracting each deviation is illustrated respectively.

MDE is defined as the largest joint displacement error for a joint in a specific exercise performed by all participants, i.e. MDE can provide insight about the magnitude of error that can be observed [21, 22]. In addition, the ROM value for shoulder abduction, elbow flexion, wrist ulnar deviation, wrist flexion, and wrist pronation were calculated to show how ROM values are affected by the joint displacement error. Fig 4 illustrates wrist pronation, flexion, and ulnar deviation.

## Results

The main focus of this study was to evaluate the stability of Kinect for measuring ROM while the body was standing in a still pose. MDE and SSE measures were considered for evaluations. Further, the stability of Kinect for 5 ROM values was evaluated.

The following abbreviation were used for better readability: K1: K2, D1: D2, P1:P13, and E1:E5, denote Kinect v1, Kinect v2, distance at 2 m form camera, distance at 3m from camera, participant 1 through 13, and exercises 1 through 5, respectively. For example, E1K1D1P1 indicates that exercise one was performed by the first participant, at the distance of 2m from the camera, captured by Kinect v1.

**Fig 5:**
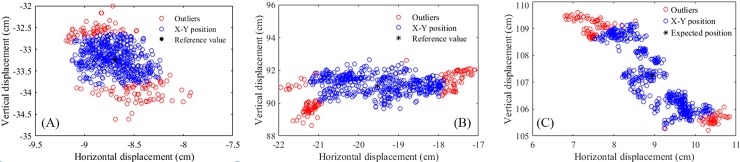
Distribution of Ankle, Hand, and Elbow in coronal plane: (A) Ankle, (B) Elbow, and (C) Hand. The X-Y values were obtained in the camera space. The black points show the reference value. Outliers are shown with red, and the blue circles indicate data points considered for stability analysis.

In order to visualize the joint displacement error, 3 examples are illustrated in Fig 5: spatial distribution of Ankle, Hand, and Elbow in XY plane with respect to the camera space. Red markers show the discarded outliers. As the figure shows, the position of joints have a large displacement error in respect to the reference value. For example, the maximum horizontal error for Ankle in E5K2D2P12 is 10mm (Fig 5A). This error for Elbow in E5K2D2P12 is 20mm in the vertical direction (Fig 5B). For Hand, the vertical error is 24mm in E5K2D2P8 (Fig 5C).

Table 1 summarizes MDE and SSE for joints that have the same poses in all exercises. In this table MDE_x,y,z_ represents the displacement errors in X,Y, and Z directions, respectively. All the reported values are calculated across all participants.

**Table 1:** Displacement error (mm) for joints that have a similar pose.

	D1K1		D1K2		D2K1		D2K2	
	SSE	MDE_x,y,z_	SSE	MDE_x,y,z_	SSE	MDE_x,y,z_	SSE	MDE_x,y,z_
Ankle-L	9.00	8,82,14	1.60	5,11,12	9.70	8,31,7	4.80	19,4,10
Ankle-R	5.40	11,80,14	1.70	7,8,12	9.90	24,33,6	5.90	27,4,8
Foot-L	14.6	22,76,53	2.10	9,4,17	13.3	16,53,46	7.10	27,25,53
Foot-R	11.1	16,74,52	1.90	5,4,14	13.5	20,57,44	8.90	28,27,59
Knee-L	7.00	13,67,17	3.90	15,7,19	7.00	38,31,45	4.60	13,6,13
Knee-R	6.90	15,51,19	4.10	15,6,21	7.30	39,46,46	5.20	31,8,41
Hip	6.80	26,5,26	5.80	22,15,26	4.90	36,18,45	3.50	31,10,26
Hip-L	6.60	24,5,26	5.80	21,18,25	5.00	34,21,45	3.50	25,11,28
Hip-R	6.60	26,4,26	5.90	23,12,26	4.80	33,16,44	3.50	30,9,24
Spine	7.00	26,4,26	6.60	25,8,28	5.00	34,23,45	4.10	28,6,27
Shoulder-C	9.30	31,19,34	7.70	26,6,31	6.90	39,46,46	4.80	31,8,41
Head	14.3	13,67,17	13.6	15,7,19	8.70	55,46,52	6.20	43,20,45

D1 and D2: distance at 2 and 3 meters from camera, K1: Kinect v1, K2: Kinect v2, SSE: Sum of Square Error, MDE_x,y,z_: Maximum Displacement Error in x, y, z directions.

Considering MDE in Table 1, the error increases with distance when comparing D1 to D2. The maximum error values occur in D2 using K1. Further, it was observed that K2 is more stable compared to K1. K1 and K2 show similar results in the hip region which includes Hip-C, Hip-R and Hip-L. The maximum errors for joints in this region are 36, 21, and 45 mm in respect to X, Y, and Z directions. The largest MDE values were observed for lower extremities which include Foot, Ankle and Knee which reach up to 82 mm.

Some notes on SSE values in Table 1 are also worth mentioning. SSE ranges vary from 5mm to 10mm for K1 and K2 for most of the joints at D1 and D2. K2 shows less sensibility to distance with respect to distance with SSE in range of [1mm 7mm]. The largest error is observed in Head and Foot for both Kinect cameras. This error can be seen up to 15mm in K1. For lower extremities, the error increases with distance when comparing D2 to D1 for both cameras. Another result that arises from Table 1 is that K2 has a smaller error in both distances over all exercises. For example, the error range decreases to more than 5mm in Ankle and Foot for K2 in comparison with K1.

Table 2 summarizes SSE for Hands, Wrists, Elbows, and Shoulders for each exercise separately, because they take a different pose in each exercise. Based on the last column of Table 2, total SSE for Shoulder and Elbow is less than 10mm for both Kinects at D1 and D2. For the rest of the joints, total SSE is between 10 to 15 mm. The average SSE for each test case is also shown in this table. In most cases SSE is less than 10 mm per test case. Considering all joints, the largest error can be observed in Hand and Wrist. For example, in E5K1D1 SSE is over 35mm. In exercises such as E4 and E5 some joints are occluded by other joints. For E5, Elbow was occluded by Hand (see Fig 2). This issue can be clearly observed in Table 2. Accordingly, Elbow and Wrist showed larger SSE values in E4 and E5 as compared to E1 through E2. The smallest total SSE was observed for Shoulder with an average SSE of 7mm.

**Table 2:** Average displacement error (mm) for Elbow, Hand, Shoulder, and Wrist joints.

		E1D1	E1D2	E2D1	E2D2	E3D1	E3D2	E4D1	E4D2	E5D1	E5D2	Total
K1	Elbow-L	8.90	5.70	6.70	4.40	12.8	6.50	13.1	11.5	17.4	12.0	9.90
	Elbow-R	9.80	5.80	6.90	6.70	11.3	7.80	13.4	9.90	13.2	8.80	9.30
	Hand-L	10.6	7.30	7.70	6.30	23.3	11.3	29.0	13.2	37.4	7.00	15.3
	Hand-R	11.4	7.30	7.30	6.90	20.0	11.7	36.0	12.5	19.1	6.70	13.8
	Shoulder-L	9.10	5.50	6.80	4.80	10.7	5.00	9.30	8.30	8.00	11.9	7.90
	Shoulder-R	9.10	5.70	6.80	4.70	9.80	5.10	8.10	6.70	8.80	8.90	7.30
	Wrist-L	10.1	6.00	7.10	5.00	19.7	9.60	23.4	14.0	30.3	9.40	13.4
	Wrist-R	11.1	6.10	7.20	6.30	15.6	10.2	28.4	13.7	15.6	7.10	12.1
Average of test case		10.0	6.20	7.10	5.60	15.4	8.40	20.0	11.2	18.7	8.97	11.1
K2	Elbow-L	8.40	5.10	7.00	4.10	12.4	7.30	9.20	8.20	13.5	9.20	8.40
	Elbow-R	8.30	5.10	6.60	3.90	11.2	7.70	9.20	8.50	12.4	10.2	8.30
	Hand-L	9.80	5.40	8.10	4.50	15.8	11.3	30.8	10.2	45.2	7.00	14.8
	Hand-R	11.0	5.80	7.40	4.10	15.0	11.0	30.1	11.7	35.4	6.90	13.8
	Shoulder-L	8.70`	4.90	7.20	4.40	8.70	4.90	6.10	5.00	11.1	3.80	6.40
	Shoulder-R	8.10	5.10	6.70	4.20	8.80	4.90	7.40	4.70	7.80	3.50	6.10
	Wrist-L	9.00	5.40	7.50	4.40	15.7	10.5	24.5	11.1	39.1	8.00	13.5
	Wrist-R	10.0	5.60	7.20	4.50	14.7	9.80	24.7	12.5	32.8	8.70	13.0
Average of test case		9.10	5.30	7.20	4.20	12.7	8.4	17.7	8.90	24.6	7.10	10.5

D1 and D2: distance at 2 and 3 meters from camera, K1: Kinect v1, K2: Kinect v2, E: exercise.

**Fig 6:**
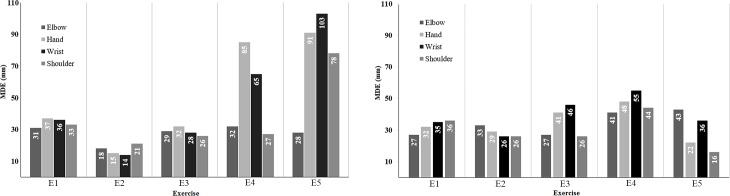
MDE errors for Elbow, Hand, Wrist, and Shoulder using (A) Kinect v2 joints at distance 1 (K2D1), and (B) Kinect v2 joints at distance 2 (K2D2).

Fig 6 shows MDE for joints mentioned in Table 2. For each joint MDE is presented for X, Y, and Z directions. The complete MDE is presented in S1 Appendix. In general, MDE for these joints is significantly larger than SSE. In most cases this error was over 20 mm. For Wrist and Hand, this error was more than 50 mm, while reaching up to 100mm in some cases. Considering SSE values, Kinect shows good stability, however, based on MDE, Kinect might result invalid measurements. As an example, Wrist has a MDE of 103mm at E5K2D2, while the SSE for this joint is 8mm.

**Fig 7:**
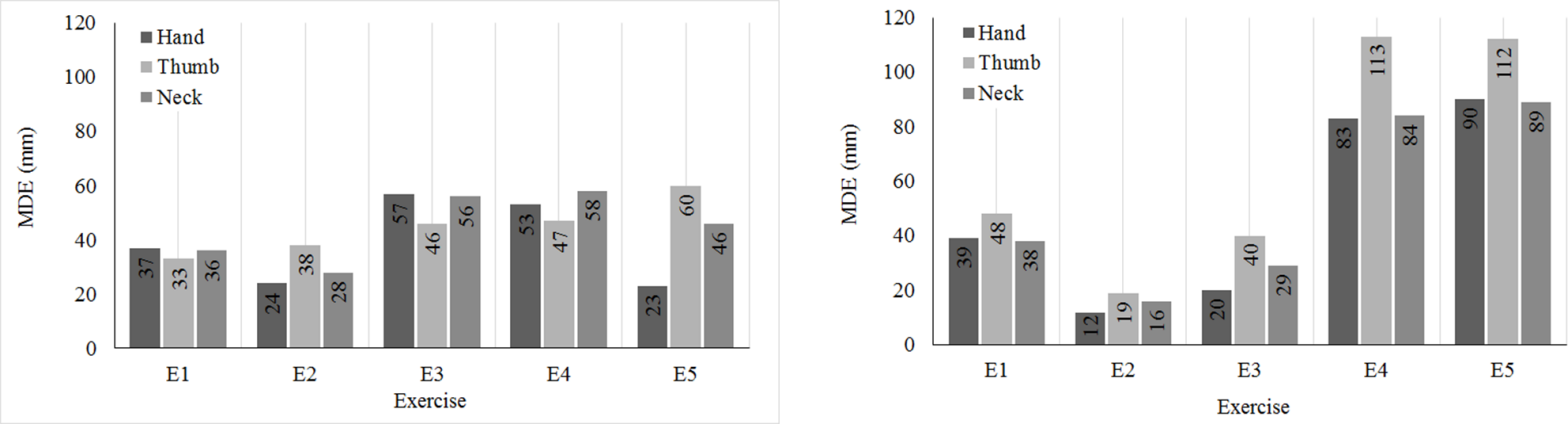
Maximum error (MDE) for additional joints provided by Kinect v2. Measurements at distance (A) D2, and (B) D1.

Table 3 summarizes the errors for Hand Tip, Neck, and Thumb. These joints are only available for Kinect v2. Hand-Tip and Thumb have relatively large displacement error. For Hand-tip the error was about 30 mm in E4 and up to 50mm for Thumb in E5. Total average error is about 15mm for both joints. Moreover, Kinect v2 has smaller error of about 6mm for Neck and it seems that measurement of this particular joint is more stable. Fig 7 illustrates MDE for Hand Tip, Thumb, and Neck. The complete MDE table is presented in S1 Appendix. The maximum errors are mostly in the range of [26mm 60mm]. Based on MDE values significant displacement errors can be expected.

**Table 3:** SSE (mm) for Hand Tip, Thumb, and Neck joints provided by Kinect v2.

	E1D1	E1D2	E2D1	E2D2	E3D1	E3D2	E4D1	E4D2	E5D1	E5D2	Total
Hand Tip-L	11.5	6.70	9.40	5.5	18.8	12.7	31.9	11.4	45.8	8.0	16.1
Hand Tip-R	12.0	7.10	8.40	5.0	17.7	12.7	33.4	12.4	33.3	8.1	15.0
Thumb-L	19.0	9.90	11.2	6.5	21.6	12.0	32.9	11.3	53.7	8.6	18.6
Thumb-R	18.8	10.3	10.1	5.5	16.9	12.1	34.0	12.7	38.6	7.7	16.6
Neck	8.90	5.40	7.60	4.6	8.80	4.90	7.60	5.80	7.40	4.6	6.50

D1 and D2: distance at 2 and 3 meters from camera, E: exercise. SSE: Some of Squared Error.

ROM values are calculated using trigonometry relations between the joints. The instability of ROM values are resulted from instable joint position delivered by Kinect. Therefore, the results were shown based on joint displacement error. Stability analysis for wrist, shoulder abduction and elbow flexion are presented in Table 4. Wrist ulnar deviation was measurable in E5, while the angle in other exercises were calculated for E3 and E4. The results are only available for K2.

**Table 4:** MAE and SSE in elbow flexion, shoulder abduction, wrist flexion, wrist pronation, and wrist ulnar deviation.

	E3D1		E3D2		E4D1		E4D2		E5D1		E5D2	
	MAE	SSE	MAE	SSE	MAE	SSE	MAE	SSE	MAE	SSE	MAE	SSE
Elbow flexion	6.19	1.31	1.58	0.94	21.74	4.11	8.04	3.24	-		-	
Shoulder abduction	4.77	0.99	2.18	0.92	9.07	1.89	8.87	1.77	-		-	
Wrist flexion	8.96	5.10	3.59	1.90	66.72	10.39	4.37	2.5	-		-	
Wrist pronation	14.50	5.40	6.41	4.51	29.46	7.25	3.63	1.76	-		-	
Wrist ulnar deviation	-		-		-		-		27.56	10.18	41.59	10.83

D1 and D2: distance at 2 and 3 meters from camera, E: exercise. MAE: Maximum Angle Error. SSE: Some of Squared.

According to the results reported in Table 4, the ROM values were measured with less than 10° variation error on average, except for the wrist pronation. However, based on Maximum Angle Error (MAE), large error variations was observed. For example, in E4 due to the occlusion of joints, noticeable variation error was seen in Elbow flexion and Wrist ROMs. Considering Table 2, it can also be observed that the Elbow has significant displacement error in E4. For shoulder abduction, smaller error was obtained when compared to other ROM values. This stability is due to the use of Hip joint in shoulder abduction measurement (Table 2). Wrist ROM, especially wrist pronation and ulnar deviations show errors of more than 10°. The results are confirmed by Table 3 where large displacement errors were found for Hand Tip and Thumb. Furthermore, it can be added that ROM measurement is directly influenced by joint displacement error.

## Discussion

ROM measurement is a common approach for quantifying motion limitations of physically disabled patients. The Microsoft Kinect which uses machine vision techniques to extract 3D joint positions may be a low-cost solution for ROM measurement. This study was conducted to evaluate the stability of Kinect for ROM measurement during static stretching exercises. The ROM values were derived by applying trigonometry rules between the Kinect joint positions.

On average, the SSE values for the joints positions was less than 15mm, however, the maximum errors in terms of MDE were significant. Kinect v2 showed to be more stable compared to the Kinect v1. Furthermore, Kinect V2 showed to be more stable considering the distance of the subject from the camera. This may be due to the significantly higher resolution depth image provided by the Kinect v2’s built in depth sensing technology which allows for more accurate joints position estimation. Furthermore, the stability of the Kinect readings were sensitive to occlusions. In exercises with body part occlusion such E4 less stable readings were observed.

**Fig 8:**
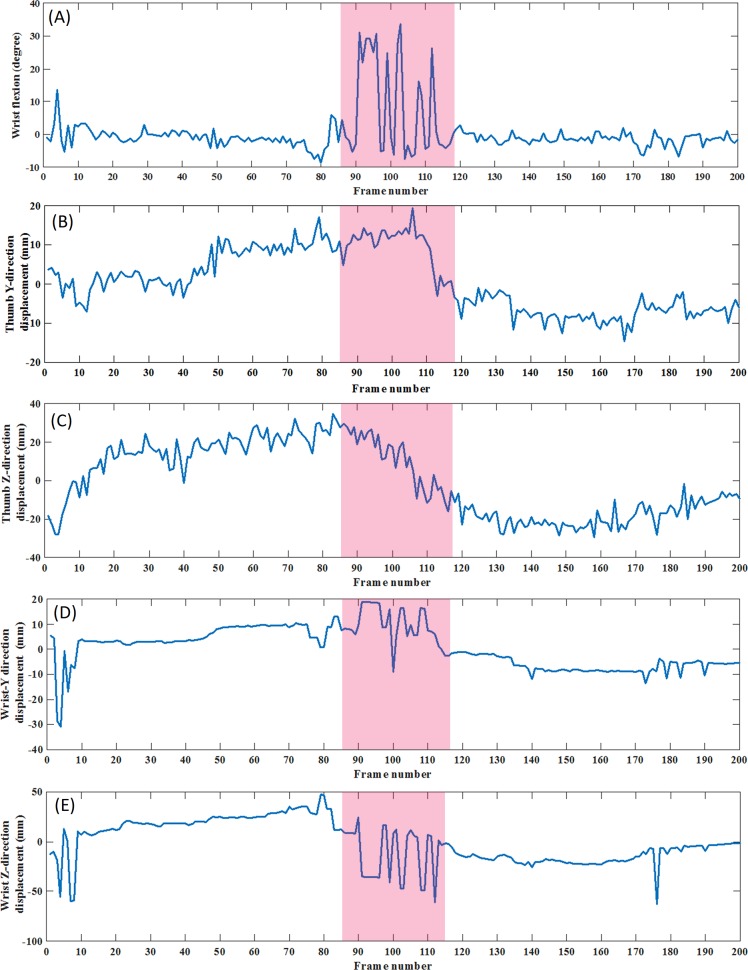
(A) Wrist pronation angle during E4K2D2P1. (B-E) Joints used to obtain wrist pronation angle. For frames colored in red, small deviation of joints position results in large deviation of the measured angle.

The average ROM values reported in Table 4 resulted in error values of less than 10° for most measurement. However, significant MDEs were observed. The ROM values were highly dependent on the stability of the joints positions. Small displacement errors of joints positions resulted in significant ROM errors. To visualize this effect, wrist flexion in E4K2D2P1 is illustrated for 200 frames (Fig 8). Wrist flexion angle was calculated based on readings for Wrist and Thumb. Figs 8B–8E show the displacement error for the mentioned joints with respect to the reference point. It can be seen that angular displacement variations for frames 90: 110 reach to more than 20°. In this study, removal of outliers and averaging of the error values were considered as simple steps for signal conditioning. In future studies, more sophisticated de-nosing techniques such as frequency domain filtering and rank filters need to be implemented and evaluated.

The number of participants in this study was limited to 13. However, the number of participants is comparable to previous studies that have evaluated Kinect for ROM assessment. For example, Bonnecher et al, [22] considered 48 participants to evaluate the validity and accuracy of Kinect v2 within functional assessment activities. Zulkarnain et al, [23] evaluated Kinect for digital data acquisition of shoulder range of motion using 10 participants. Wang et al, [12] recruited 10 participants to evaluate Kinect v1 and Kinect v2 for pose tracking accuracy. Reither et al [15], measured the accuracy of Kinect for ROM measurement using 1 participant in two sessions with four repetitions. Regarding the number of samples per exercise, a static exercise is recommended to be held for 10 to 30 seconds [24]. Our recording sessions were also designed so that each exercise was recorded for at least 10 seconds.

The exercises chosen in this study were limited to movements along anatomical body planes. However, the source of instabilities are the incorrect estimation of the joint position which is extracted on frame-by-frame basis. For marker-less motion capture systems these errors are mostly independent of the exact motion [23].

The results of this study have implications for researchers and clinicians who intend to use Kinect for ROM assessment. Based on the results of this study, the following points need to be considered if using Kinect as a ROM assessment device: The setup procedure of the camera may impact the outcome measurements. For lower-body joints, results showed that Kinect is more stable when the subject is standing 2m away from the camera. More stable readings of the upper-body joints were obtained when subject was standing 3m from the camera. MDE error also revealed that signal processing techniques such averaging filters and appropriate frame selection should be considered to provide more stable readings when using Kinect for ROM measurement.

## Conclusion

The stability of Kinect v1 and v2 was evaluated for ROM measurements stability during static stretching exercises. 5 static exercises were performed by 13 volunteers at 2 distances from the camera. The detected skeleton in each frame was captured and joints position were recorded for data analysis. MDE and SSE were applied to assess the stability of Kinect. Overall observation showed that Kinect v2 generally provides more stable result compared to Kinect v1. SSE error for most of the joints was less than 10mm, however, MDE values were larger than 50mm for many of the joints. Distance from camera and joint occlusion during an exercise affects the stability of Kinect. Based on MDE values, large displacement errors are possible for some individual test cases. Pre-processing and signal enhancements techniques such as de-noising and appropriate frame selection should be considered for improving the stability of Kinect for rehabilitation applications.

## Supporting information

S1 Appendix This file include the complete MDE tables. (DOCX)
