# Supplementary material for: Stability of Kinect for range of motion analysis in static stretching exercises
Source: PLoS One. 2018 Jul 24;13(7):e0200992. doi: 10.1371/journal.pone.0200992 (PMC6057630; doi:10.1371/journal.pone.0200992)
Supplement: S1 Appendix — (DOCX) [file pone.0200992.s001.docx]

Appendix

Table 1. MDEx,y,z for Elbow, Hand, Shoulder, and Wrist

|  | | E1D1 | E1D2 | E2D1 | E2D2 | E3D1 | E3D2 | E4D1 | E4D2 | E5D1 | E5D2 |
| --- | --- | --- | --- | --- | --- | --- | --- | --- | --- | --- | --- |
| K1 | Elbow-L | 24 ,7,30 | 35,31,28 | 12,4,11 | 10,5, 31 | 35 ,8 ,19 | 11,17,28 | 19, 11,35 | 33,35,54 | 17, 10,47 | 14,39,64 |
|  | Elbow-R | 33 ,8, 32 | 29,14,42 | 8, 4,14 | 12,45,29 | 29 ,9 ,17 | 37,16,27 | 13 ,15,33 | 17,32,44 | 18, 10, 25 | 24,10,22 |
|  | Hand-L | 16,13,30 | 42,19,34 | 13,5,13 | 15,24,31 | 47,21,29 | 35,29,40 | 74, 32,39 | 13,50,51 | 48,57,128 | 16, 17,17 |
|  | Hand-R | 46,16,33 | 31,17,43 | 8, 8, 11 | 23,60,29 | 44,24,19 | 56,31,28 | 97,79,48 | 18,52,74 | 20,12, 41 | 15, 13,18 |
|  | Shoulder-L | 27 ,5 ,28 | 38 ,5,33 | 9 ,3,14 | 20 ,8 ,35 | 29 ,5,26 | 15 ,5,25 | 12,10,30 | 15,19,54 | 11, 7,23 | 26, 57,33 |
|  | Shoulder-R | 28 ,4, 33 | 37, 6,38 | 7 ,3, 16 | 19 ,4, 25 | 29, 4,23 | 17,6 ,27 | 12 ,6 ,33 | 31,13,45 | 7 ,15,21 | 13, 47,20 |
|  | Wrist-L | 17,10,29 | 34, 9,32 | 13,4,12 | 13,12,33 | 43,13,22 | 24,23,36 | 55,25,39 | 29,40,50 | 34,45,98 | 16, 19,52 |
|  | Wrist-R | 41,12,32 | 28,11,46 | 9 ,6 ,12 | 12,43,31 | 46,12,17 | 56,31,28 | 87,63,44 | 28,48,43 | 21,12,26 | 17, 12,16 |
|  | |  |  |  |  |  |  |  |  |  |  |
| K2 | Elbow-L | 22 ,6,31 | 27,14,26 | 9 ,6 ,18 | 8 ,16 ,33 | 27,9,17 | 16,22,27 | 15 ,10 , 32 | 9 ,29,41 | 25, 15, 28 | 14,13, 43 |
|  | Elbow-R | 25 ,7,33 | 25 ,8 ,39 | 7, 5, 15 | 7 ,7 , 25 | 29,10,14 | 41,26,26 | 9 , 12 , 29 | 14,30,50 | 20 ,13, 16 | 41,15, 49 |
|  | Hand-L | 14, 9, 28 | 28,6 ,32 | 11,5,15 | 7 , 9 ,29 | 24,14,24 | 41,41,28 | 109,175,52 | 14,48,40 | 115,174,58 | 17,18, 22 |
|  | Hand-R | 37,12,33 | 28,15,35 | 8 ,8, 10 | 8 ,10,25 | 32,16,18 | 34,52,28 | 85 ,53 ,69 | 16,50,63 | 75 ,91 ,75 | 14,20, 23 |
|  | Shoulder-L | 20 ,6, 31 | 32, 5,23 | 6, 4, 21 | 12,13,31 | 26,6 ,20 | 11, 6,24 | 11 ,3 , 27 | 17,12,41 | 70 ,78 ,15 | 10, 4 ,18 |
|  | Shoulder-R | 22 ,3 ,33 | 33 ,5,36 | 6 ,5, 19 | 10,6, 26 | 28, 8,22 | 12,4 ,26 | 17 ,5 ,27 | 8 ,13,44 | 43 , 9, 16 | 6 , 5 , 16 |
|  | Wrist-L | 15,10,29 | 28,12,29 | 10,5,14 | 7 ,7 ,32 | 25,12,22 | 38,39,29 | 80,133, 46 | 18,46,42 | 88,133 ,48 | 20 ,25 ,25 |
|  | Wrist-R | 34,11,36 | 25,11,35 | 12,9,12 | 18,25,26 | 31,13,16 | 28,46,28 | 65, 41, 35 | 16,48,55 | 58 ,82 ,103 | 11 ,31 ,36 |
|  | |  |  |  |  |  |  |  |  |  |  |

| Table 2. MDE_x,y,z_ for Hand Tip, Neck, and Thumb | | | | | | | | | | |
| --- | --- | --- | --- | --- | --- | --- | --- | --- | --- | --- |
|  | E1D1 | E1D2 | E2D1 | E2D2 | E3D1 | E3D2 | E4D1 | E4D2 | E5D1 | E5D2 |
| Hand Tip-L | 16,8,32 | 29,10,33 | 12,6,16 | 8,10,29 | 24,18,26 | 44,46,29 | 109,174,51 | 17,52,42 | 114,173,57 | 14,22,20 |
| Hand Tip-R | 39,14,32 | 28,12,37 | 8,9,12 | 10,8,24 | 19,20,20 | 40,57,32 | 83,52,69 | 17,53,46 | 74,90,47 | 13,23,21 |
| Neck | 36,29,38 | 27,25,36 | 12,16,12 | 13,12,28 | 29,16,19 | 35,56,29 | 84,52,66 | 58,48,47 | 73,89,82 | 46,30,23 |
| Thumb-L | 21,6,33 | 33,8,39 | 13,6,21 | 13,8,26 | 27,3,21 | 12,4,24 | 14,7,32 | 6,6,45 | 17,7,20 | 7,6,23 |
| Thumb-R | 20,28,48 | 33,27,32 | 19,10,15 | 38,36,34 | 28,31,40 | 36,46,29 | 109,113,52 | 43,47,44 | 112,109,56 | 60,18,23 |
